# Solids Turn into Liquids—Liquid Eutectic Systems of Pharmaceutics to Improve Drug Solubility

**DOI:** 10.3390/ph15030279

**Published:** 2022-02-23

**Authors:** Mafalda C. Sarraguça, Paulo R. S. Ribeiro, Cláudia Nunes, Catarina Leal Seabra

**Affiliations:** 1 LAQV-REQUIMTE, Faculdade de Farmácia, Universidade do Porto, Rua de Jorge Viterbo Ferreira, 228, 4050-313 Porto, Portugal; cdnunes@ff.up.pt (C.N.); cseabra@ff.up.pt (C.L.S.); 2 Centro de Ciências Sociais, Saúde e Tecnologia, Universidade Federal do Maranhão, Imperatriz 65900-410, Brazil; pauloufv@hotmail.com

**Keywords:** eutectic systems, therapeutic liquid eutectic systems, diabetes, solubility, green chemistry, chlorpropamide, tolbutamide

## Abstract

The low solubility of active pharmaceutical ingredients (APIs) is a problem in pharmaceutical development. Several methodologies can be used to improve API solubility, including the use of eutectic systems in which one of the constituents is the API. This class of compounds is commonly called Therapeutic Deep Eutectic Systems (THEDES). THEDES has been gaining attention due to their properties such as non-toxicity, biodegradability, and being non-expensive and easy to prepare. Since the knowledge of the solid liquid diagram of the mixture and the ideal eutectic point is necessary to ascertain if a mixture is a deep eutectic or just a eutectic mixture that is liquid at ambient temperature, the systems studied in this work are called Therapeutic Liquid Eutectic Systems (THELES). Therefore, the strategy proposed in this work is to improve the solubility of chlorpropamide and tolbutamide by preparing THELES. Both APIs are sulfonylurea compounds used for the treatment of type 2 diabetes mellitus and have low solubility in water. To prepare the THELES, several coformers were tested, namely, tromethamine, L(+)-arginine, L-tryptophan, citric acid, malic acid, ascorbic acid, and *p*-aminobenzoic acid, in molar ratios of 1:1 and 1:2. To improve viscosity, water was added in different molar ratios to all systems. THELES were characterized by mid-infrared spectroscopy (MIR), and differential scanning calorimetry. Their viscosity, solubility, and permeability were also determined. Their stability at room temperature and 40 °C was accessed by MIR. Cytocompatibility was performed by metabolic activity and cell lysis evaluation, according to ISO10993-5:2009, and compared with the crystalline APIs. THELES with TRIS were successfully synthesized for both APIs. Results showed an increased solubility without a decrease in the permeability of the APIs in the THELES when compared with the pure APIs. The THELES were also considered stable for 8 weeks at ambient temperature. The cells studied showed that the THELES were not toxic for the cell lines used.

## 1. Introduction

Commonly, Deep Eutectic Systems (DES) are defined as eutectic mixtures in which the depression of the melting point of the mixture is such that the mixture is liquid at room temperature [1,2].

DESs are gaining widespread scientific and technological attention due to their advantages. They are recognized as less volatile, thermally stable, biodegradable, less toxic, low in cost, and highly tunable due to the amount of different species that can constitute them [3,4,5]. Additionally, the preparation of DESs is conducted according to Green Chemistry principles, e.g., 100% atom economy, without the formation of by-products, and therefore they can be considered green solvents and alternatives to organic solvents and ionic liquids [3,6]. DESs are normally comprised of a hydrogen bond acceptor (HBA) and a hydrogen bond donor (HBD). Strong noncovalent interactions between the HBA and HBD, such as hydrogen bonds, alkyl–alkyl interactions, halogen bonds, and Van der Walls forces are the basis for the depression of the melting point and at the same time inhibition of crystallization [7].

DESs have been studied in a variety of fields of the chemical, pharmaceutical, biotechnology, and electrochemical industries. In pharmaceutical applications, DESs have been used to design polymeric drug delivery systems [8,9], to deliver poorly water−soluble drugs [10,11,12], for dermal and transdermal drug delivery [13,14], and to design active pharmaceutical ingredient-based DESs [15,16,17].

Most of the active pharmaceutical ingredients (APIs) may act as HBDs or HBAs and can, therefore, be used as DES phase–forming. In this case, they are called Therapeutic Deep Eutectic Systems (THEDES), and in their constitution, one or both constituents are pharmaceutical relevant molecules [1,2,5,18,19,20]. THEDES can be obtained by combining the API with a variety of compounds depending on the aim, for example, metabolites [21] or permeation enhancers [13,22]. The constituents of THEDES are molecules whose toxicity profiles are well known and are already used in pharmaceutical applications, which presents an advantage in comparison with ionic liquids.

The low solubility of drugs, in particular new drugs, is still a major problem in the pharmaceutical industry and one of the major causes of failure of new drug candidates [23]. A large variety of strategies has been developed over the years to overcome the solubility problem, among them the use of salts [24], co-crystals [25,26,27,28], and co-amorphous systems [29,30,31]. In the last few years, the use of THEDES to improve solubility and permeability of drugs belonging to classes II and IV of the Biopharmaceutical Classification System (BCS) has been increasing. A few examples are the mixture of paeonol with menthol for transdermal delivery with enhanced skin permeation [32], and the eutectic mixture of ibuprofen and menthol with an increase of ibuprofen solubility by 12 fold [19]. The solubility increase of the API was also studied for the eutectic mixture of citric acid:ethambutol:water (2:1:10), and for the mixture of citric acid:L-arginine:water in several ratios. For the THEDES with ethambutol, the solubility increase was 27.5-fold; for the systems with arginine, a small increase in the solubility was found [33].

The presence of water in the eutectic system can be a way to manipulate the viscosity in a non-toxic way [34]. Water has been described to act as a second small HBD when the eutectic mixture contains 1 to 2 mol of water by mol of DES [35]. However, when the amount of water is above this threshold, no clear picture of the systems has been defined. It has been suggested that water contributes to the slightly ordered HB network formed among DES components; however, these studies used chlorine cations [35,36]. López-Salas studied the effect of the addition of water in DESs [37]. This study showed that water has a role as an additional HBD or HBA; moreover, they found out that the systems containing water had a lower depression of the melting point compared with the same system without water, suggesting that a controlled addition of water can be used to obtain deeper eutectics. They also showed that the addition of water gave rise to a much lower viscosity DES compared with the original one, preserving the most features of this sort of solvent formed by H-bond complexes and with a lower melting point.

The definition of DES and THEDES that is present in most of the literature may not be the most correct. According to Coutinho et al. [38], for a DES to be different from a eutectic mixture, the eutectic temperature point must be below that of the ideal liquid mixture [38]. The presence of a eutectic point in a solid–liquid equilibrium phase diagram is a characteristic of all mixtures that are fully or partially immiscible in the solid phase [38]. These authors studied some systems that are called DES and found out that in most cases the eutectic point does not deviate from the ideal one, and therefore they should be called only eutectic mixtures. From a thermodynamic perspective and according to some authors, a DES should be defined as a mixture of two or more pure compounds in which the eutectic point temperature is the bellow of that of an ideal liquid mixture, presenting significant negative deviations from ideality. Additionally, the temperature depression should be such that the mixture is liquid at operating temperature for a certain temperature range” [38].

In this paper, and since we did not study the ideal behavior of the mixtures, we call these systems Therapeutic Liquid Eutectic Systems (THELES) instead of THEDES. Independently of the name, the fact is that these systems have properties that could be very helpful in pharmaceutical development, especially their capacity to dissolve APIs.

Chlorpropamide and tolbutamide are oral hypoglycemics that belong to the sulfonylureas class and are largely used in the treatment of Type II diabetes mellitus, a chronic disease that generates disturbances in the use and production of insulin in the organism. These APIs have limiting factors due to their low solubility in water that limits their bioavailability [39,40].

In this work, a tri-component system comprised of the API (chlorpropamide and tolbutamide), a coformer, and water were studied with the possibility to form THELES. The THELES were characterized by mid-infrared (MIR) spectroscopy and differential scanning calorimetry (DSC); their viscosity was determined, as well as their solubility and stability at 25 °C and 40 °C. Cytocompatibility was performed by metabolic activity and cell lysis evaluation, and then cell permeability was assessed using Transwell^®^ cultures.

## 2. Results

To synthesize the THELES, several coformers were tested, namely, tromethamine (TRIS), L-arginine (ARG), citric acid (CIT), malic acid (MAL), ascorbic acid (ASC), *p*-aminobenzoic acid (PABA), and tryptophan (TRY). Water was added to all systems in a defined ratio. From the combinations of API and coformers tested (Table 1), only the combination with tromethamine (TRIS) and water resulted in THELES. Water was needed to have a liquid eutectic mixture at ambient temperature, as preliminary tests showed that without water the mixture was solid. The THELES with CLP and TLB are, from now on, designated by CLP-THELES and TLB-THELES, respectively.

**Figure 1:**
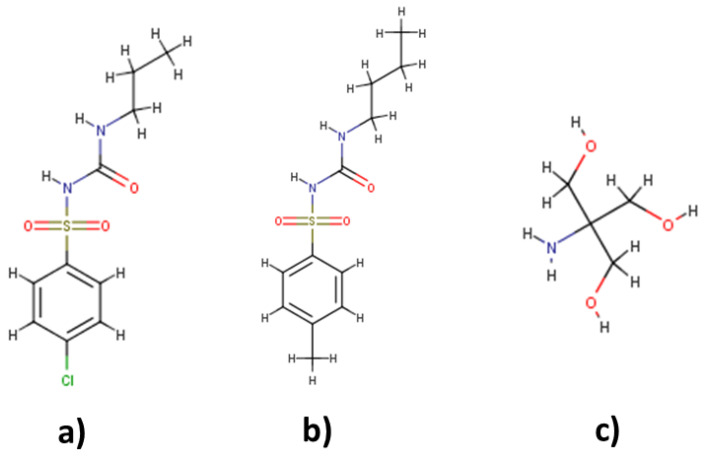
Structures of chlorpropamide (CLP) (**a**), tolbutamide (TLB) (**b**), and tromethamine (TRIS) (**c**).

From the ratios of API:TRIS:H_2_O tested, the ratios that were proven to be stable for at least one week at room temperature were CLP:TRIS:H_2_O (1:2:6) and TLB:TRIS:H_2_O (1:2:8). The percentage of water in weight present in each THELES was 17.23% and 21.95% for CLP-THELES and TLB-THELES, respectively. These combinations of API:TRIS:H_2_O formed a mixture that was liquid and transparent at room temperature. Therefore, these two THELES were further characterized and studied to evaluate their suitability for formulation development. The structures of the APIs and TRIS can be found in Figure 1.

### 2.1. Thermal Behavior and Vibrational Spectra

**Figure 2:**
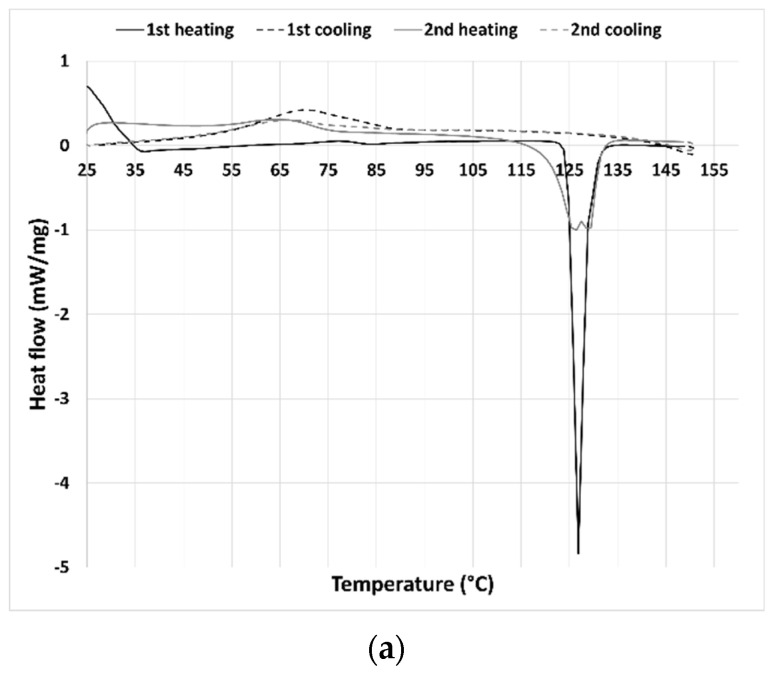

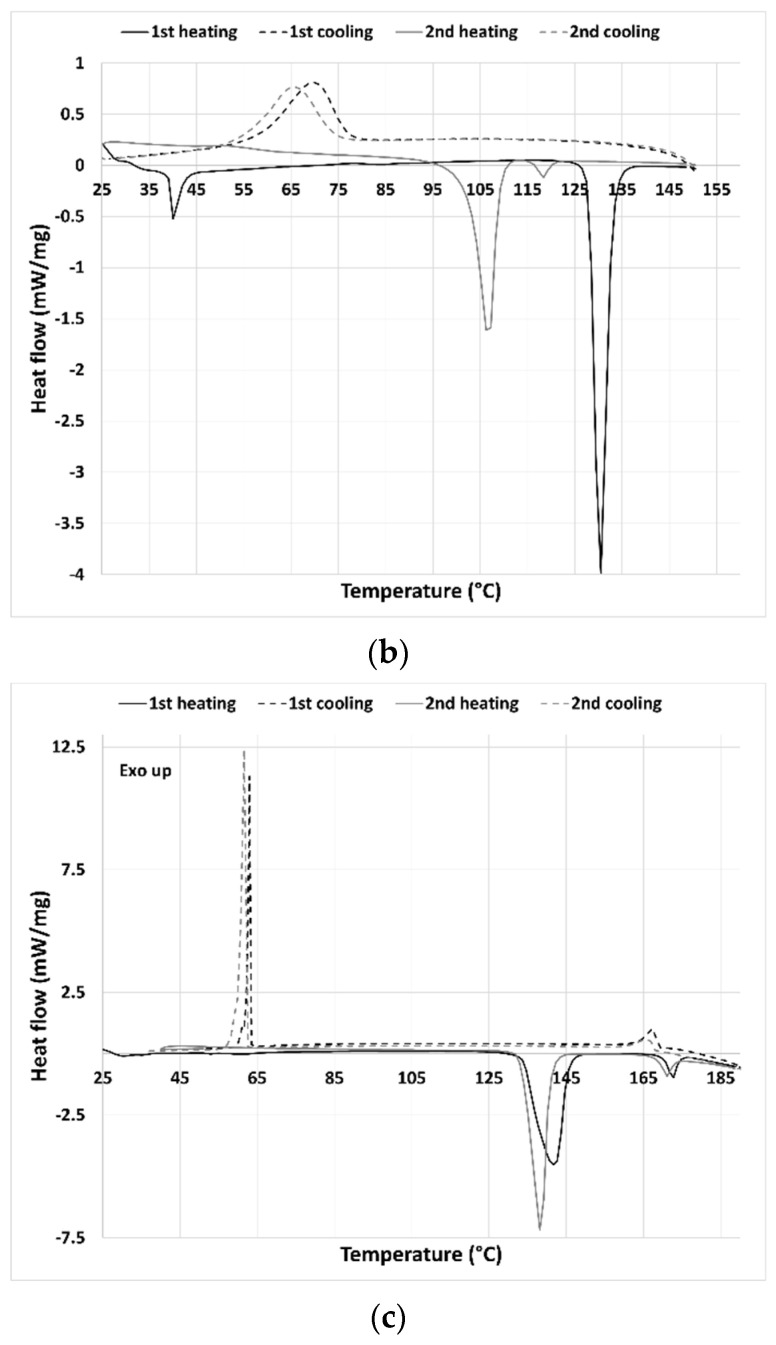
DSC thermograms from cooling/heating cycles from (**a**) pure CLP, (**b**) pure TLB, and (**c**) pure TRIS.

**Figure 3:**
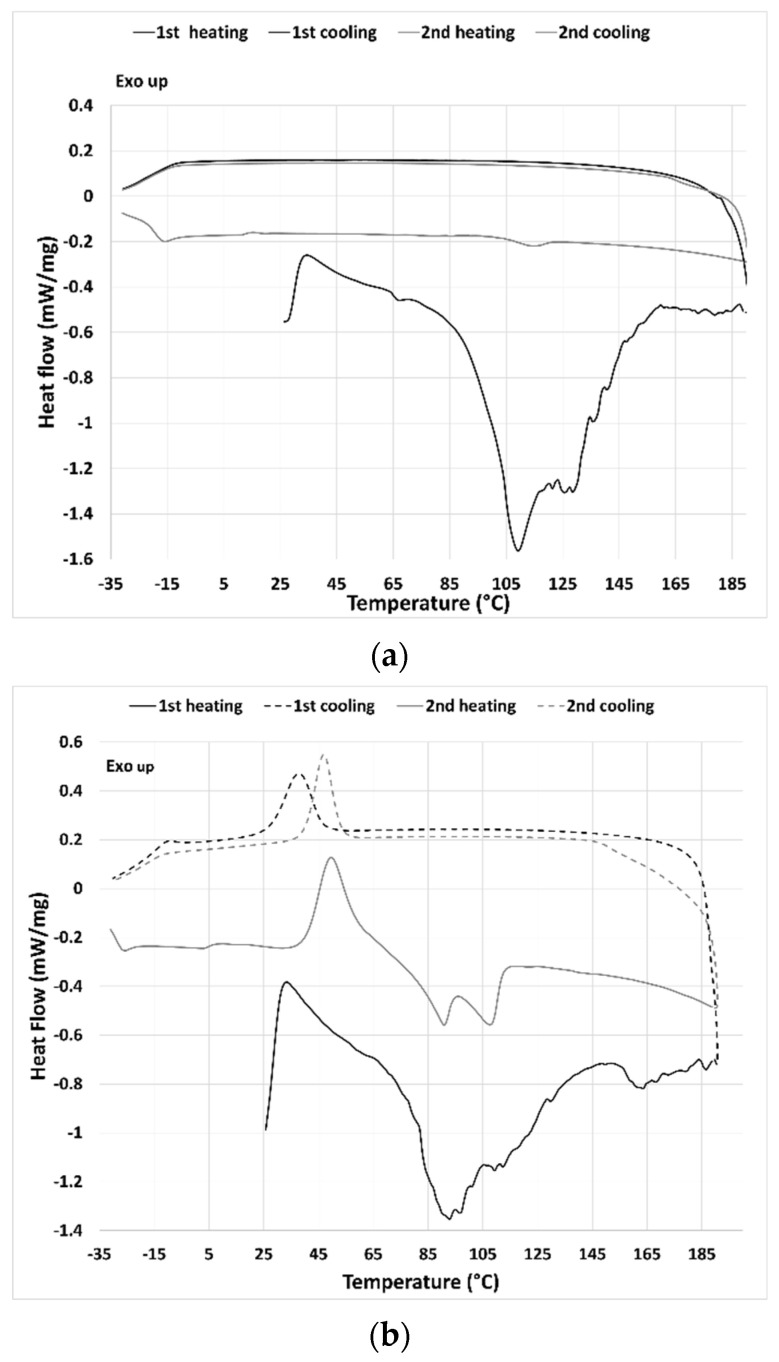
DSC thermograms from cooling/heating cycles from (**a**) CLP-THELES and (**b**) TLB—THELES.

The thermal behavior of the synthesized THELES and the parent compounds was investigated by DSC by conducting two heating/cooling cycles (Figure 2 and Figure 3). For CLP (Figure 2a) the first heating run showed an endothermic event corresponding to the melting with an onset temperature of 124.9 °C. Upon cooling, a broad exothermic peak could be perceived with an onset temperature of 54.3 °C. Chlorpropamide is known to exhibit at least six polymorphic phases and very complex thermodynamic relationships [40]. In the second heating run, two endothermic events could be seen with a peak temperature of 126.1 °C and 129.0 °C. Calorimetric studies showed that one of the polymorphs of CLP (α-chlorpropamide) transformed at heating into another polymorph (polymorph C) [41]. The double peak seen in the thermogram is the melting of the two polymorphs.

Tolbutamide has four polymorphs known (I–IV) [39]. The tolbutamide thermogram (Figure 2b) showed two endothermic events in the first heating run; the first event at 39.2 °C corresponded to a solid–solid transition between polymorphic forms I^L^ and I^H^; and the second one to the melting of form I^H^ at 128.1 °C [39]. In the cooling run, an exothermic event can be seen with an onset temperature of 54.1 °C, corresponding to the TLB crystallization. In the second heating run, an endothermic peak with an onset of 103.0 °C can be seen that according to the literature corresponds to the transition between form III to I^H^ [39]; however, no melting peak of form I^H^ can be seen in the thermogram. The second endothermic event had an onset temperature of 116.5 °C and was coincident with the melting temperature of form II [39].

The tromethamine thermogram (Figure 2c) presented in the first and second heating run an endothermic event at 134.0 °C, corresponding to a solid–solid transition (from α to γ form) and a melting peak at 170.3 °C [42]. Both cooling runs had two exothermic events at around 162 °C and 61 °C that may correspond to the crystallization of both polymorphic forms.

The thermal behavior of the THELES can be seen in Figure 3. For CLP-THELES (Figure 3a) in the first heating run, a large endothermic event was present with what seemed to be two peaks at ~109 °C and ~128 °C. The first event was due to the water evaporation, and the second to the melting of CLP. In both cooling cycles, no thermal event could be perceived, and in the second heating run, a small event that started at ~105 °C could be seen. It is difficult to understand the nature of the event; however, it is likely a glass transition since no crystallization was seen in the previous cooling run.

In the first heating run of the TLB-THELES (Figure 3b), three endothermic peaks could be seen at ~93 °C, ~112 °C, and 163 °C. The first peak may have been due to water loss; however, it was difficult to define the second and third peaks, since they were not close to any of the thermal events from the initial components. In the first cooling run, a crystallization peak could be seen with an onset temperature of 27.7 °C. In the second heating run, a glass transition with a glass transition temperature (Tg) at −15 °C could be perceived followed by a cold crystallization with an onset of 40.5 °C followed by two endothermic events with peak temperatures of 90.0 °C and 107.7 °C.

**Figure 4:**
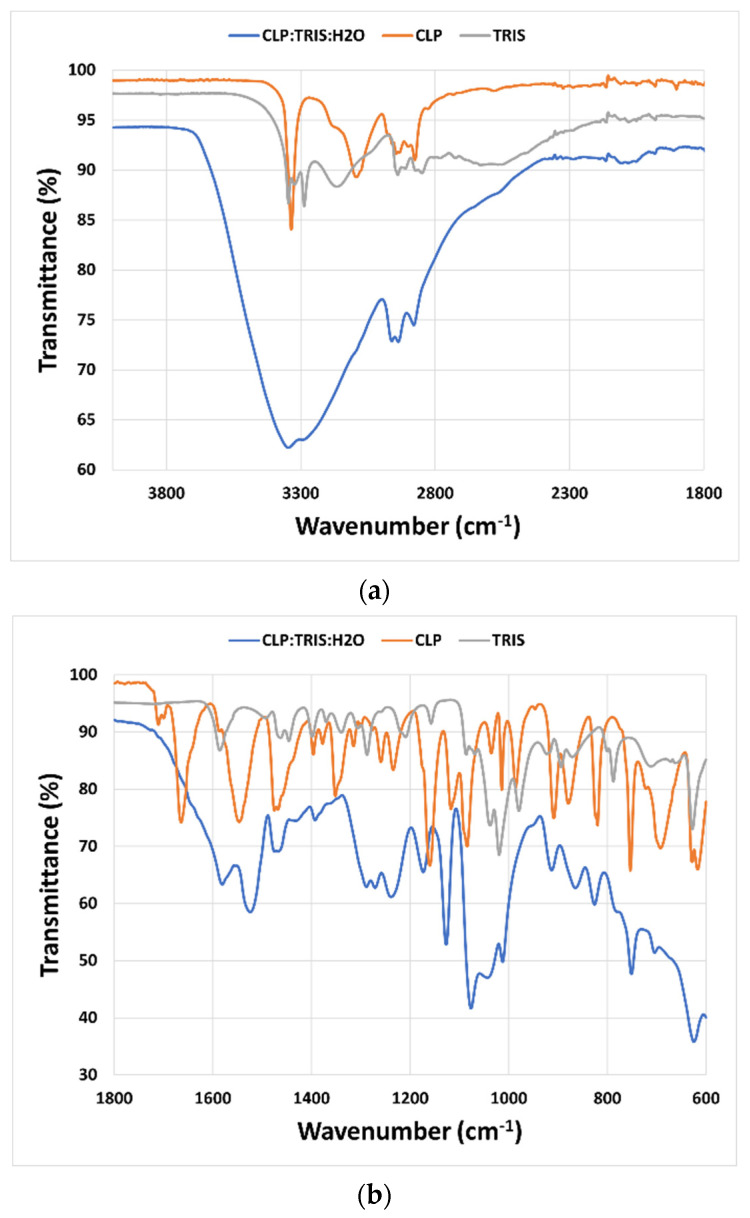
Mid-infrared spectra from the CLP-THELES pure crystalline CLP and pure crystalline TRIS. (**a**) Spectral region between 4000 cm^−1^ and 1800 cm^−1^; (**b**) spectral region between 1800 cm^−1^ and 600 cm^−1^.

**Figure 5:**
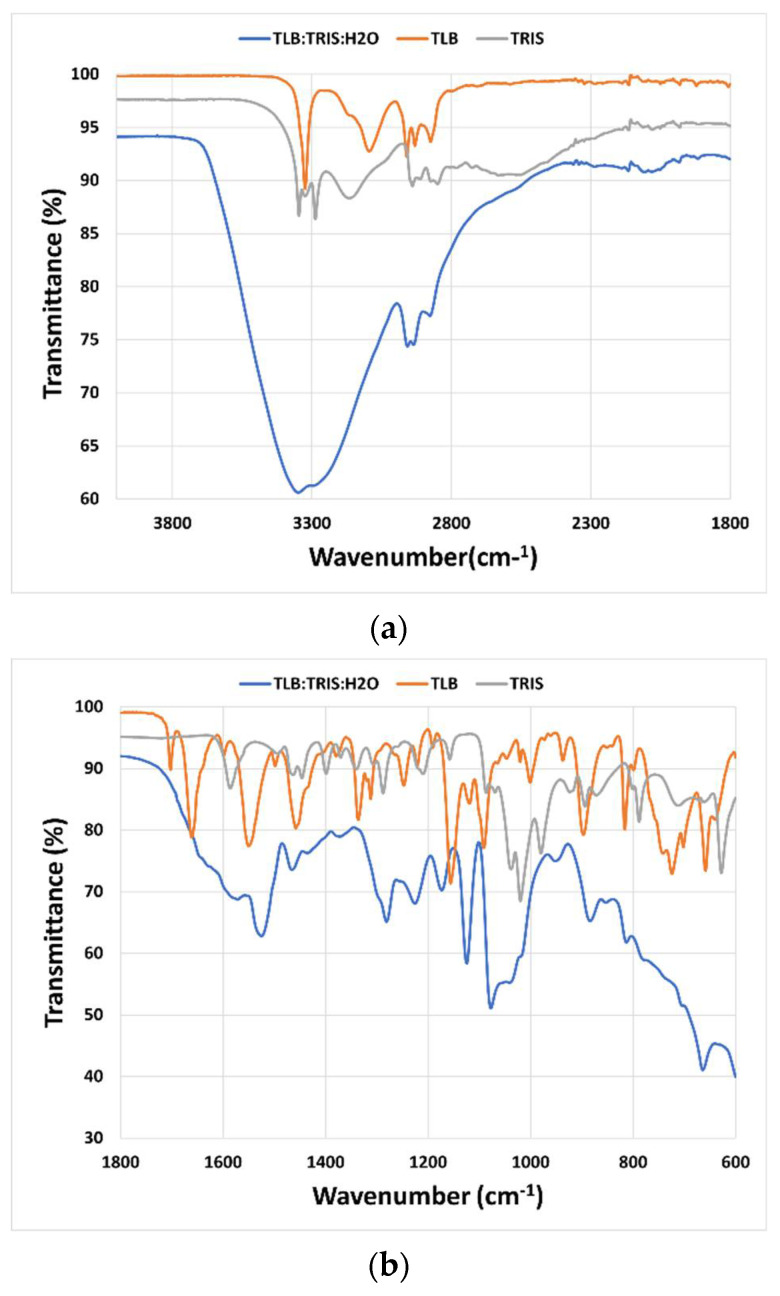
Mid infrared spectra from the TLB-THELES, pure crystalline TLB, and pure crystalline TRIS. (**a**) Spectral region between 4000 cm^−1^ and 1800 cm^−1^; (**b**) spectral region between 1800 cm^−1^ and 600 cm^−1^.

Mid-infrared (MIR) spectroscopy was used to study the interactions between the molecules that constitute the THELES (Figure 4 and Figure 5). The spectral region between 4000 cm^−1^ and 1800 cm^−1^ did not give much information due to the OH stretching vibration band of water around 3350 cm^−1^ that overwhelmed this spectral region. In the fingerprint region, several differences could be seen when comparing the spectra of the THELES and the spectra of the starting materials. In the spectra from the CLP THELES (Figure 4), the bands from the vibration of NH (1586 cm^−1^) and CO (1020 cm^−1^) of TRIS and the bands of the carbonyl group (1665 cm^−1^), NH (1546 cm^−1^), and the S=O bands (1352 cm^−1^, 1160 cm^−1^, 1082 cm^−1^) of CLP were the ones that presented major changes. However, the region between 1800 cm^−1^ and 1600 cm^−1^ was also difficult to analyze due to the bending vibration band of water at around 1600 cm^−1^.

Similar spectral changes could be seen in the THELES with TLB (Figure 5), since the two APIs have similar structures (see Figure 1).

### 2.2. Stability Studies

The stability was accessed at room temperature and 40 °C by mid-infrared spectroscopy for nine weeks. At room temperature for CLP-THELES after week 8, some spectral differences were visible due to water loss (Figure S1). This could be clearly perceived by changes in the OH vibration band of water at 3350 cm^−1^. Additionally, spectral features due to the NH vibrations of TRIS could be seen in the spectrum from week 8 onwards. For TLB-THELES (Figure S2), no major spectral differences could be seen during the entire period of analysis. At 40 °C for the CLP-THELES, after week 4, spectral changes associated with loss of water could be already noticed (Figure S3). For the TLB-THELES, the loss of water was observed notably after week 6 (Figure S4).

### 2.3. Viscosity, Solubility, and Cell Permeability

**Figure 6:**
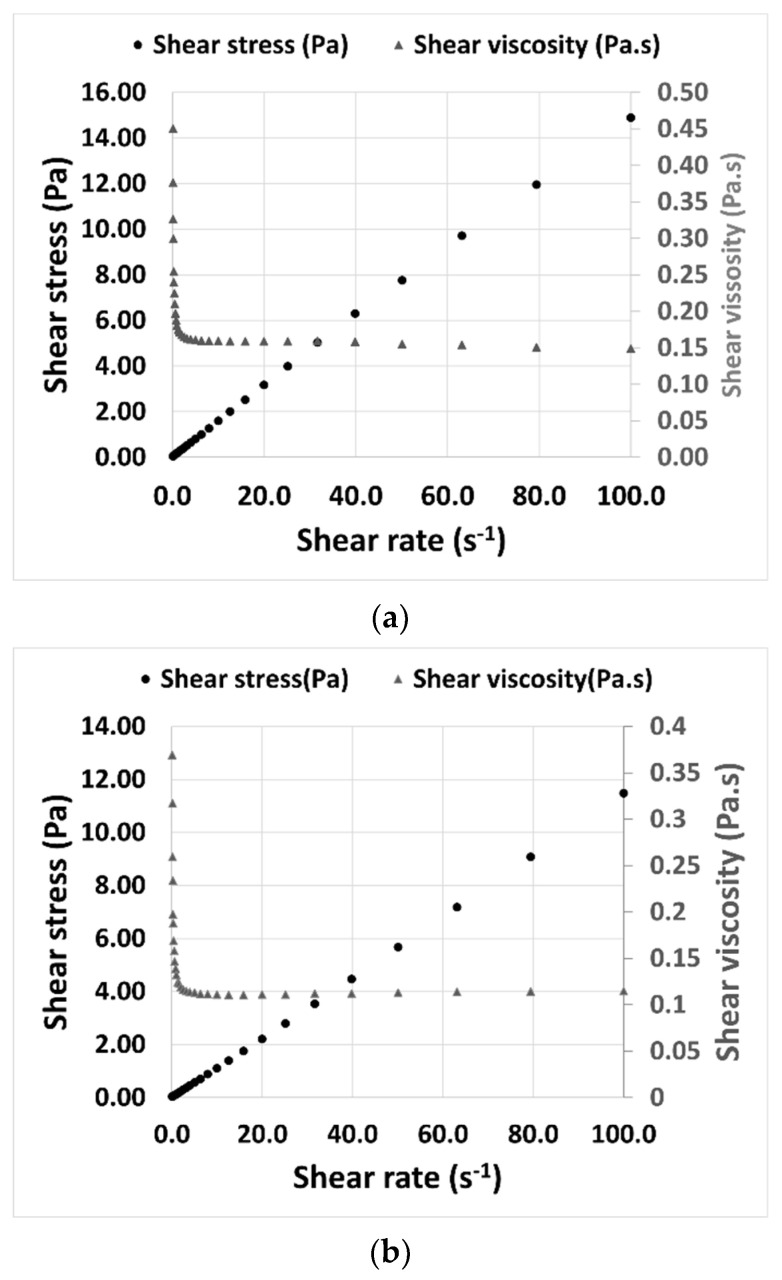
Shear stress and shear viscosity versus shear rate for the (**a**) CLP-THELES and (**b**) TLB-THELES.

The viscosity of THELES was determined at 25 °C to understand its rheological behavior. Both THELES behaved as a Newtonian fluid, since there was a constant proportionality between the shear stress and the shear rate (Figure 6). The viscosities stabilized around 0.15 Pa.s and 0.11 Pa.s for the CLP-THELES and TLB-THELES, respectively.

**Figure 7:**
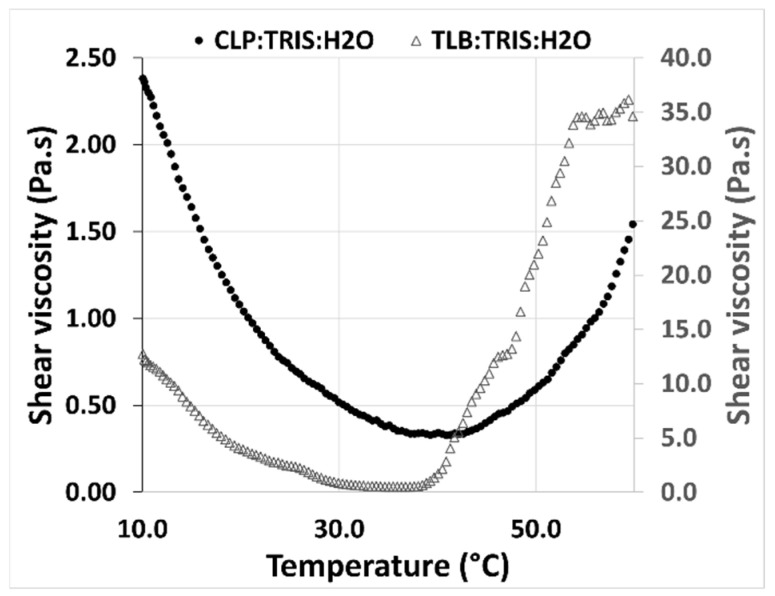
Shear viscosity in the function of temperature for the CLP-THELES and TLB-THELES.

The rheological behavior of the THELES with temperature was studied between 10 °C and 60 °C (Figure 7). For both THELES, the viscosity decreased with temperature until a certain temperature around 40 °C and 35 °C for CLP-THELES and TLB-THELES, respectively.

**Figure 8:**
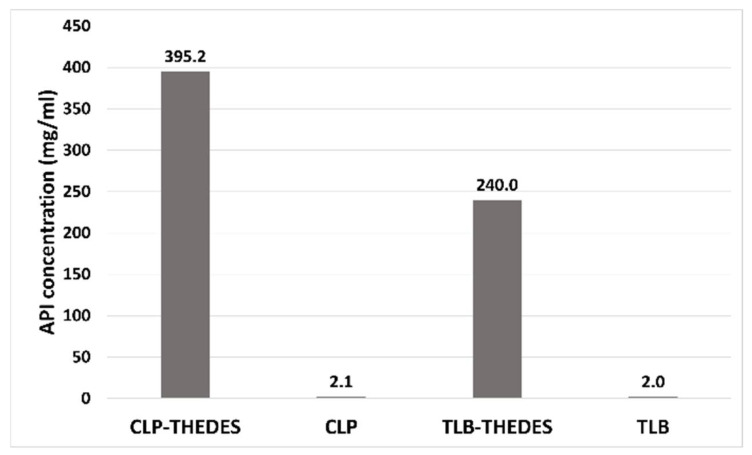
Solubility of the THELES and the crystalline APIs in PBS (pH = 7.4) buffer.

The solubility was determined in phosphate buffer (pH = 7.4). The solubility of both APIs in the THELES was much higher than the respective crystalline API (Figure 8). For chlorpropamide, the increase was 188 times, from 2.1 mg·mL^−1^ of crystalline CLP to 395 mg·mL^−1^ for CLP in the THELES. In the case of tolbutamide, the increase was from 2 mg·mL^−1^ to 240 mg·mL^−1^, thereby increasing 120 times.

**Figure 9:**
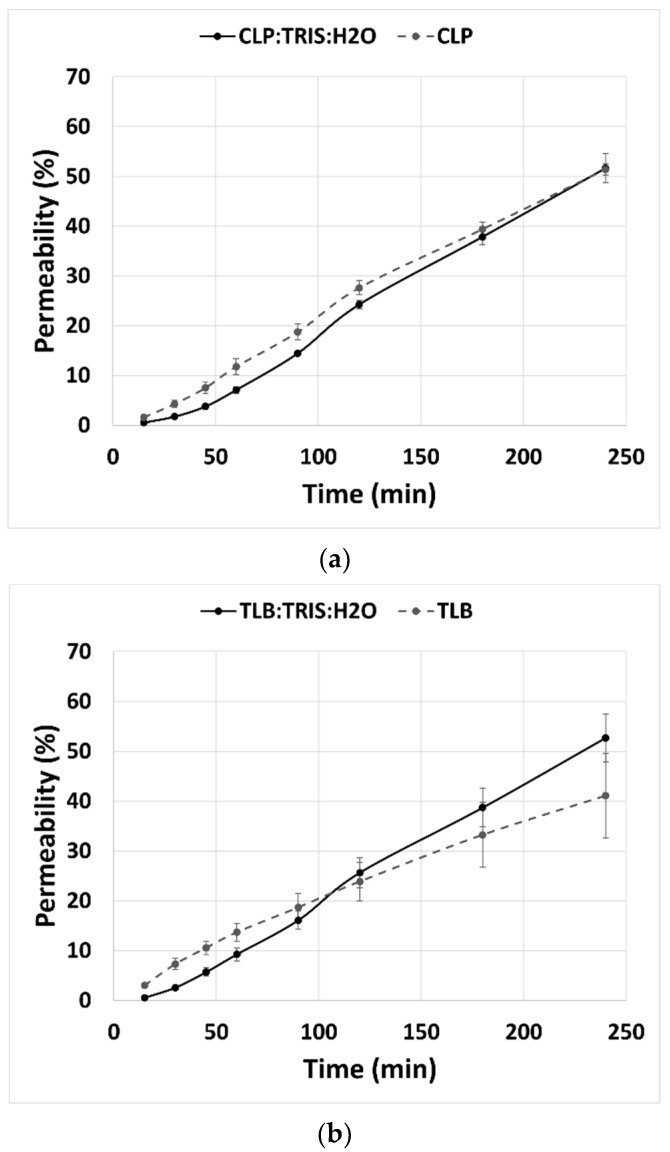
Percentage of permeability for (**a**) CLP-THELES and (**b**) TLB-THELES.

Permeability studies were done for 240 min with eight sampling points. Results showed that the percentage of permeability of CLP and TLB in the THELES had the same profile, along the 240 min, as the percentage of permeability of the crystalline APIs (Figure 9). At 240 min, the percentage of permeability for crystalline CLP and CLP-THELES was 51.4% and 51.6%, respectively. For TLB, the percentage of permeability was 41.1% and 52.7% for crystalline TLB and THELES-TLB, respectively.

### 2.4. Cytocompatibility of THELES

**Figure 10:**
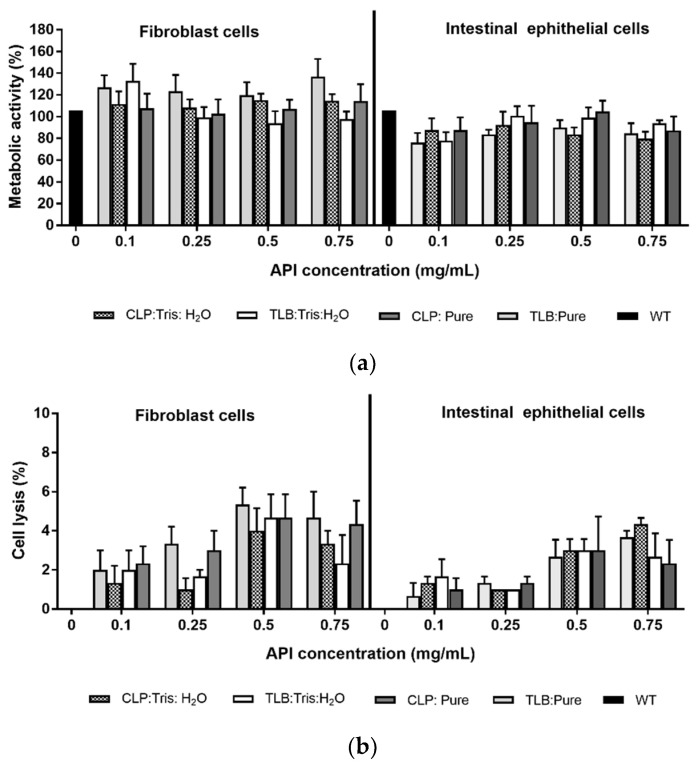
(**a**) Metabolic activity and (**b**) cell lysis for the THELES and crystalline APIs using fibroblast and intestinal epithelial cell lines. WT means wild-type, untreated cells (control).

Figure 10 shows the effect of THELES and pure APIs on fibroblast cells and intestinal epithelial cells viability and lysis determined by MTT (Figure 10a) and LDH (Figure 10b) assays, respectively. For the concentrations tested, there was not a significant reduction (values higher than 70%) in the cell metabolic activity in THELES and pure APIs. In fact, for fibroblast cells, there were values above 100%, suggesting that these compounds favor cell growth.

Regarding the effect of the THELES in cell lysis, no toxicity was observed for the THELES, and pure APIs (values lower than 30%), for all concentrations tested, with values lower than 8% of cell lysis (Figure 10b).

## 3. Discussion

Improvement of the physicochemical properties of APIs, namely its solubility and therefore its bioavailability, is of the utmost importance for pharmaceutical development. Water low solubility is one of the main elements that restrict the usage of many potential APIs. Insoluble drugs in the gastrointestinal tract cause slow drug release rates and generally exhibit erratic and incomplete absorption [43]. CLP and TLB have low solubility in water and high permeability; therefore, low solubility is the factor that hinders their bioavailability. Therapeutic liquid eutectic systems (THELES) have been recognized as improving drug solubility [19]; therefore, this work aimed to develop THELES of CLP and TLB to improve their solubility. From the several coformers tested, the best results were obtained with tromethamine (TRIS). TRIS is an amine proton acceptor (HBA) [42], and therefore in these THELES, the APIs are the proton donors (HBD). In recent years, there has been growing interest in TRIS as a promising pharmaceutical excipient because it is possible to increase the dissolution rate of drugs, alter membrane permeability, increase the bioavailability of sparingly soluble drugs, and increase drug stability [42].

The pKa of each of CLP, TLB, and TRIS is 5.13, 5.16, and 8.08, respectively; therefore, there is the possibility of having a proton transfer from the APIs to TRIS. The pH of the THELES was measured for both samples, and it was 8.60 and 9.0 for CLP-THELES and TLB-THELES, respectively. At this pH, both APIs are deprotonated, the hydrogen of the sulfonamide is lost, and the amine of TRIS is protonated. With the techniques employed to characterize the THELES, we cannot confirm that this occurs. The changes that could occur in the MIR spectra are obscured by the presence of the water bands. Therefore, we can only say there is a possibility that these systems are protic ionic liquids and not molecular liquids. This possibility also explains the high increase of the solubility of both APIs [44,45].

Both THELES are Newtonian fluids (Figure 6) characterized by a linear relationship between the shear stress and the shear rate [46]. The viscosity value for CLP-THELES is higher, even if slightly, than the value for TLB-THELES, since the API molecules are very similar; therefore, the difference in the viscosity is mainly due to the amount of water present, which is higher in the TLB-THELES, leading to the decrease of the viscosity. This behavior was already studied by other authors [47]. However, the presence of water within these systems is reported to change physicochemical properties, which could influence molecular interactions with APIs [48]. In one case, the authors studied the behavior of a DES with and without the addition of water and found the incorporation of water distorted the structure of the DES due to the incorporation of an additional bond donor or acceptor into the hydrogen bond complex. Additionally, the authors found that introduction of water resulted in a depression of the eutectic temperature by the formation of a new eutectic [37]. These results suggest that the introduction of water in the DES or THELES can enhance the system performance by reducing the eutectic temperature.

The study of the rheological behavior with temperature (Figure 7), showed a decrease of the viscosity with the increase of temperature. This behavior is linked with the decrease in the magnitude of H-bonding with temperature, and it is a normal behavior of DESs [49]. However, after a certain temperature (40 °C for CLP-THELES and ~37 °C for TLB-THELES), the viscosity starts to increase with temperature. This increase can be related to the loss of water resulting in a more viscous fluid. This assumption can be corroborated by the DSC results (Figure 3) in which a large endothermic event due to water loss can be seen in the thermograms. The stability studies showed that at ambient temperature, both THELES are stable at least for 8 weeks, and that at 40 °C the stability is reduced for 4 and 6 weeks for CLP-THELES and TLB-THELES, respectively. These results combined demonstrated that both THELES are stable until temperatures close to the human body temperature (37 °C).

The interaction between the components of THELES was studied by MIR; however, the presence of water makes it difficult to interpret spectral regions important to understand the bonds between the components, since the band due to the O–H vibration of water (~3500 cm^−1^) overwhelms the spectral region between 4000 cm^−1^ and 1800 cm^−1^ (Figure 4a and Figure 5a). This spectral region has information regarding the stretching vibrations of the O–H and N–H bonds, which are the synthons normally involved in the non-covalent bonds between the compounds [27]. The fingerprint region (Figure 4b and Figure 5b) gives some information regarding the main groups involved in the formation of the eutectic mixture. The N–H and the C–O vibration bands of TRIS and the C=O, N–H, and S=O vibration bands suffered alterations in the spectra for both THELES. However, the spectral region that includes the carbonyl and the amide group of CLP and the amine group of TRIS is also difficult to interpret, since water is also a band at 1600 cm^−1^. The sulphonamide group of CLP and TLB is clearly involved in the formation of the eutectic mixture. This group is very electronegative and has been recognized as an important synthon in, for example, cocrystal formation [25,26].

CLP and TLB belong to class II of the Biopharmaceutical Classification System (BCS), meaning that they have low solubility and high permeability [40,50]. THELES have been used to increase the solubility and permeability of drugs, and even if in some cases the solubility did not increase, the dissolution profile was enhanced, since the THELES are liquids at room temperature [2,19]. For the THELES studied in the paper, the solubility was increased by 188 times for CLP and 120 times for TLB; additionally, the permeability did not change when compared with the pure APIs. Therefore, both APIs can be considered BCS class I drug (high solubility and high permeability).

For any new pharmaceutical ingredient, toxicity profiling is extremely important, and even if the initial components are considered non-toxic, THELES can have different toxicity profiles [51]. The effect of water in DES in the toxicity towards human cancer cells was studied by Hayyan et al., showing that there was not an increase in the toxicity of the studied DES [52]. In this work, two types of cell lines were used to determine the cytotoxic profile of the THELES. Results showed that the THELES have a metabolic activity similar to the pure APIs and near 100%, and that an increase of cell lysis higher than 30% was not observed, the value defined in ISO 10993-5 [53] for cytotoxic materials. Therefore, the THELES can be considered safe for human consumption and can be further used in pharmaceutical formulations.

## 4. Materials and Methods

### 4.1. Materials

Chlorpropamide (CLP, >97% purity), tolbutamide (TLB, >97% purity), ascorbic acid (ASC, >99.0% purity), citric acid (CIT, ≥99.5% purity), *p*-aminobenzoic acid (PABA, 99.0% purity), tromethamine (TRIS, >99.0% purity), Triton^®^ X-100, thiazolyl blue tetrazolium (MTT), dimethyl sulfoxide (DMSO), and Dubelcco’s phosphate buffered saline, trypan-blue solution were acquired from Sigma-Aldrich (St. Louis, MO, USA). L-(+)-arginine (ARG, ≥98% purity) was purchased from Acros Organics (Fair Lawn, NJ, USA), L-tryptophan (TRY, 99% purity) from Alfa Aesar (Kandel, Germany), and malic acid (MAL, >99.5% purity) from Merck (Darmstadt, Germany). The water used was ultra-pure grade (resistivity of 18.2 MΩ·cm), purified by a Heal Force ultra-pure water system (Shanghai, China). All chemicals were used without further purification.

Dulbecco’s modified Eagle’s medium (DMEM) with Glutamax and 4-(2-hydroxyethyl)-1-piperazineethanesulfonic acid, trypsin-EDTA (1×), penicillin–streptomycin (PenStrep), inactivated fetal bovine serum (FBS), and non-essential amino acids were purchased from Gibco by Life Technologies (New York, NY, USA. A lactate dehydrogenase (LDH) cytotoxicity detection kit was obtained from Takara Bio Inc. (Shiga, Japan). Murine fibroblast (L929) and intestinal epithelial (Caco-2) cell lines were acquired from the European Collection of Authenticated Cell Cultures (ECACC, Salisbury, UK).

### 4.2. THELES Preparation

The amount of 500 mg of the API and the respective molar amount of the coformer and water (Table 1) were weighed into a glass flask and introduced in a shaking incubator at 60 °C and 300 rpm during 10 h. After, the vials were allowed to slowly return to the ambient temperature before further analysis.

### 4.3. Mid-Infrared Spectroscopy

The samples produced were characterized by mid-infrared (MIR) spectroscopy. Spectra (*n* = 3) were acquired using a Frontier spectrometer (PerkinElmer, Beaconsfield, UK) equipped with an attenuated total reflectance (ATR) accessory (PerkinElmer, Beaconsfield, UK) equipped with a pressure arm to control the applied force and reduce sample-to-sample variability. The spectrometer had a deuterated triglycine sulfate (DTGS) detector and a mid-infrared light source. The samples were placed directly on the crystal of the ATR accessory, and each spectrum was the resultant of 32 accumulations at a resolution of 8 cm^−1^ over the range 4000–600 cm^−1^. The background spectrum was acquired for the empty crystal following cleaning.

### 4.4. Differential Scanning Calorimetry

DSC measurements were performed using a thermal analyzer (DSC 200 F3 Maia^®^, Netzsch, GmbH, Waldkraiburg, Germany) with an automatic sample changer (ASC, Netzsch, GmbH, Waldkraiburg, Germany). Approximately 7–10 mg were weighed in an aluminum pan and then sealed. The reference pan was left empty. Heating curves for the samples were recorded with a heating rate of 10 °C min^−1^. Thermograms were analyzed using the software provided by the DSC equipment (Proteus, version 6.1081, Netzsch, GmbH, Waldkraiburg, Germany). The results presented are the average of at least two measurements.

### 4.5. Rheological Studies

The rheological properties of the THELES were determined by a rotational rheometer (Kinexus lab+, Malvern, Worcestershire, United Kingdom). For viscosity measurements, a shear rate method (0.1 to 100.0 s^−1^, 10 samples per decade, at 25 °C) was used. To study the temperature effect, a single frequency temperature ramp was used with an initial temperature of 10 °C and a final temperature of 60 °C, with a 1 °C·min^−1^ ramp, and a frequency of 1 Hz. All analysis was conducted with a plate-plat configuration (geometry PU20 SR4367) with a 1 mm gap (Peltier Plate Cartridge). Data were collected using rSpace software^®^ (Kinexus, version 1.75: PSS0211-17).

### 4.6. Stability

The stability was determined by MIR using the conditions described in Section 2.3. THELES were placed in a sealed glass vial at room temperature and 40 °C away from light for 9 weeks. The spectral analysis was made once a week by retrieving an aliquot of the material that was not returned to the vial.

### 4.7. Solubility

An amount of THELES correspondent to 2000 mg of the API was added to 2 mL of PBS (pH = 7.4) and stirred for 24 h. An aliquot was taken, filtered in a 0.5 µm filter, and analyzed by UV/VIS (Jasco V-660, Tokyo, Japan) at 229 nm for CPL and 226 nm for TLB. Before this analysis, calibration curves were made using the crystalline APIs dissolved in PBS (pH = 7.4).

### 4.8. Cytocompatibility Evaluation

#### 4.8.1. Cell Culture Conditions

To assess the cytocompatibility of the API and THELES, cytotoxicity studies were performed using the murine fibroblast cell line (L929, passage 7–14) and intestinal epithelial (Caco-2, passage 10–15) cell line as recommended by the ISO10993–5 [53]. L929 cells were cultured in DMEM supplemented with 10% (*v*/*v*) FBS and 1% (*v*/*v*) PenStrep, and Caco-2 cell lines were cultured with the same medium as L929 supplemented with 1% (*v*/*v*) non-essential amino acids. Cells were incubated at 37 °C and under 5% CO_2_ in humidified air. Cells were cultured for 24 h of incubation and trypsinized when 80–90% confluence was reached, followed by counting in a Neubauer chamber with a trypan blue solution (0.4% *w*/*v*) at a ratio of 1:1. Cells were seeded at a concentration of 1 × 10^5^ cells/well to tissue culture 96-well plates (OrangeScientific^®^, Braine-l’Alleud, Belgium) and incubated at 37 °C, 5% CO_2_, for 24 h. Then, the culture medium was removed, and compounds were added to the first well at a ratio of 1:1, and serial dilutions were performed from a concentration of 750 µg/mL to 10 µg/mL. The plates were incubated for 24 h at 37 °C and under 5% CO_2_ in humidified air.

#### 4.8.2. Cell Lysis and Metabolic Activity Evaluation

The effect of API and THELES compounds in cell lines was assessed using resazurin and LDH assays. After 24 h of exposure of compounds to cells, plates were centrifuged at 250 *g* for 10 min at RT (Centrifuge 5810R, Eppendord^®^, Hamburg, Germany), and the supernatant was collected to quantify the LDH using the LDH cytotoxicity kit, according to the manufacturer’s instructions. The absorbance was read at 490 and 690 nm, using a microplate reader (Cytation 5 Cell Imaging Reader, Software: Gen5 v1.08.4, BioTeK-Instruments, Winooski, VT, USA).

The cell lysis (%) was calculated by the ratio of mean absorbance (Abs) between cells treated with 2% Triton^TM^ X-100 (positive control) and untreated cells (WT, negative control) (Equation (1)).

(1) $$\mathrm{Cell}\;\mathrm{lysis}\;\left(\%\right)=\frac{{\mathrm{Abs}}_{\mathrm{sample}}-{\mathrm{Abs}}_{\mathrm{negative}\;\mathrm{control}}}{{\mathrm{Abs}}_{\mathrm{positive}\;\mathrm{control}}-{\mathrm{Abs}}_{\mathrm{negative}\;\mathrm{control}}}\times 100$$

The MTT assay was performed to evaluate the metabolic activity of cells after 24 h of exposure to compounds. Briefly, after supernatant removal, 0.5 mg/mL of MTT was added to the wells and incubated for 2 h, at 37 °C, 5% CO_2_. Afterward, the MTT solution was removed, and dimethyl sulfoxide (DMSO) was added to dissolve the formazan crystal formed. The absorbance was measured at 550 and 650 nm, using a microplate reader (Cytation 5 Cell Imaging Reader, Software: Gen5 v1.08.4, BioTeK-Instruments, Winooski, VT, USA). Positive and negative controls were also performed using untreated cells and cells treated with 2% (*v*/*v*) Triton^TM^ X-100. The percentage of metabolic activity was calculated by the ratio of the mean absorbance (Abs) between treated (negative control) and untreated cells (WT, positive control) (Equation (2)).

(2) $$\mathrm{Metabolic}\;\mathrm{activity}\;\left(\%\right)=\frac{{\mathrm{Abs}}_{\mathrm{sample}}-{\mathrm{Abs}}_{\mathrm{negative}\;\mathrm{control}}}{{\mathrm{Abs}}_{\mathrm{positive}\;\mathrm{control}}-{\mathrm{Abs}}_{\mathrm{negative}\;\mathrm{control}}}\times 100$$

### 4.9. Cell Permeability Study

Human epithelial cells (Caco-2 cell line, passage 10–15) were seeded onto polycarbonate 24-well Transwell^®^ inserts with 3.0 μm pore size (Costar^®^, Boston, MA, USA) at the density of 1 × 10^5^ cells/insert in supplemented DMEM culture medium. Every 3–4 days, the medium was renewed, and after 24 days of culture, the transepithelial electrical resistance (TEER) was measured using an epithelial voltammeter (EVOM2, World Precision Instruments Inc, Sarasota, FL, USA) to evaluate the tight junctions formations. TEER values > 200 Ω·cm^2^ [54] were used for the permeability studies.

For the permeability studies, cells were washed twice with PBS (0.1 M, pH 7.4), and PBS was added to the correspondent inserts and receptor wells. Then, 1 mg/mL of API and THELES were added to the upper compartment (apical well). At different time-points (15, 30, 45, 60, 90, 120, 180, 240 min), 500 μL of supernatant from the lower compartment (basal well) was collected and transferred to UV 96-well plates. The concentration of compounds was measured by absorbance at a wavelength 210 nm using a microplate reader (Cytation 5 Cell Imaging Reader, Software: Gen5 v1.08.4, BioTeK-Instruments, Winooski, VT, USA), and API and THELES concentrations were calculated using a calibration curve prepared with a concentration range between 0.008 and 0.2 mg/mL in PBS, with a correlation coefficient of R^2^ = 0.9979 for CLP and of R^2^ = 0.9993 for TLB.

## 5. Conclusions

In this work, THELES from the low solubility drugs chlorpropamide and tolbutamide were successfully synthesized with tromethamine as a coformer and water as a third element. The THELES was shown to be stable at ambient temperature and temperatures near the body temperature. The APIs’ solubility was significantly increased, and the permeability was maintained when compared with the pure APIs. The cytocompatibility findings showed that THELES are safe for human cell lines, without showing a negative effect on the metabolic activity nor an enhancement of cell lysis.

These promising findings suggest that these new pharmaceutical forms can be used for further pharmaceutical development, solving the APIs’ low solubility problem and therefore limited bioavailability.

## Figures and Tables

**Table 1:** Coformers and molar ratios between the APIs, and coformers and water tested to produce the THELES. X = 2, 4, 6, 8, 10.

API	Coformer	Molar Ratio (API:Cof:H_2_O)
CLP/TLB	TRIS	1:1:X
CLP/TLB	TRIS	1:2:X
CLP/TLB	ARG	1:1:6
CLP/TLB	ARG	1:1:8
CLP/TLB	ARG	1:1:10
CLP/TLB	ARG	1:2:8
CLP/TLB	CIT/MAL/ASC/PABA/TRY	1:1:8
CLP/TLB	CIT/MAL/ASC/PABA/TRY	1:1:10

CLP—chlorpropamide; TLB—tolbutamide; TRIS—tromethamine; ARG—L(+)-arginine; TRY—L-tryptophan; CIT—citric acid; MAL—malic acid; ASC—ascorbic acid; PABA—*p*-aminobenzoic acid.

## Data Availability

Data is contained within the article and Supplementary Material.

## Supplementary Materials

The following are available online at https://www.mdpi.com/article/10.3390/ph15030279/s1, Figure S1: MIR spectra for the CLP-THELES made for 9 weeks (once a week) to study its stability at room temperature (a) spectral range between 4000 cm^−1^ and 1800 cm^−1^; (b) spectral range between 1800 cm^−1^ and 600 cm^−1^; Figure S2: MIR spectra for the TLB-THELES made for 9 weeks (once a week) to study its stability at room temperature (a) spectral range between 4000 cm^−1^ and 1800 cm^−1^; (b) spectral range between 1800 cm^−1^ and 600 cm^−1^; Figure S3: MIR spectra for the CLP-THELES made for 9 weeks (once a week) to study its stability at 40 °C (a) spectral range between 4000 cm^−1^ and 1800 cm^−1^; (b) spectral range between 1800 cm^−1^ and 600 cm^−1^; Figure S4: MIR spectra for the TLB-THELES made for 9 weeks (once a week) to study its stability at 40 °C (a) spectral range between 4000 cm^−1^ and 1800 cm^−1^; (b) spectral range between 1800 cm^−1^ and 600 cm^−1^.
