# Supplementary material for: Solids Turn into Liquids—Liquid Eutectic Systems of Pharmaceutics to Improve Drug Solubility
Source: Pharmaceuticals (Basel). 2022 Feb 23;15(3):279. doi: 10.3390/ph15030279 (PMC8951776; doi:10.3390/ph15030279)
Supplement: Supplementary file 1 [file pharmaceuticals-15-00279-s001.zip › pharmaceuticals-1511219-supplementary.pdf]

Supporting Information

Solids turn into liquids. Liquid Eutetic Systems of pharmaceutics to improve drug solubility

Mafalda Sarraguça, Paulo R. S. Ribeiro Cláudia Nunes, Catarina Leal Seabra

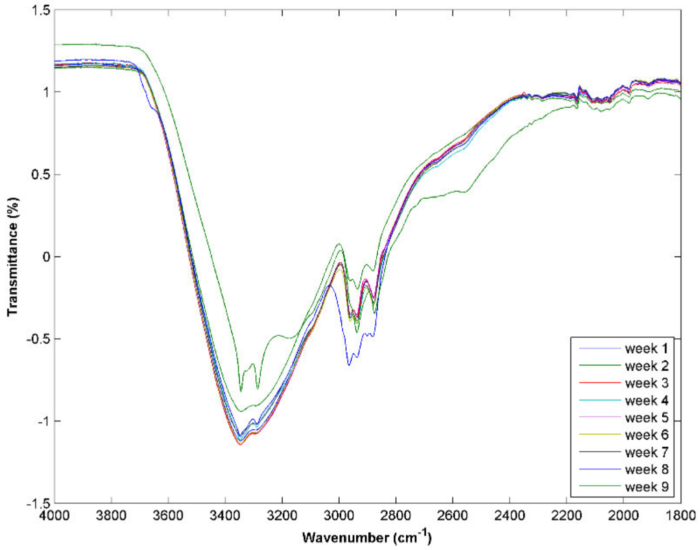

(a)

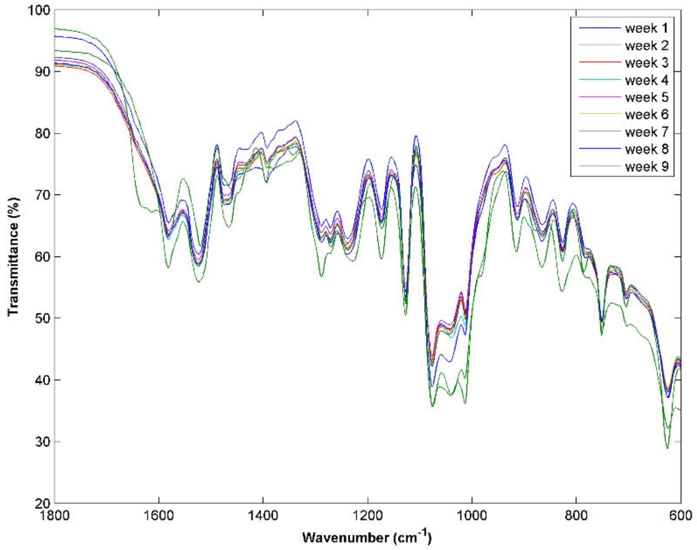

(b)

Figure *S1.* MIR spectra for the CLP-THEDES made for 9 weeks (once a week) to study its stability at room temperature **(a)** spectral range between 4000 cm^{-1} and 1800 cm^{-1}; **(b)** spectral range between 1800 cm^{-1} and 600 cm^{-1}.

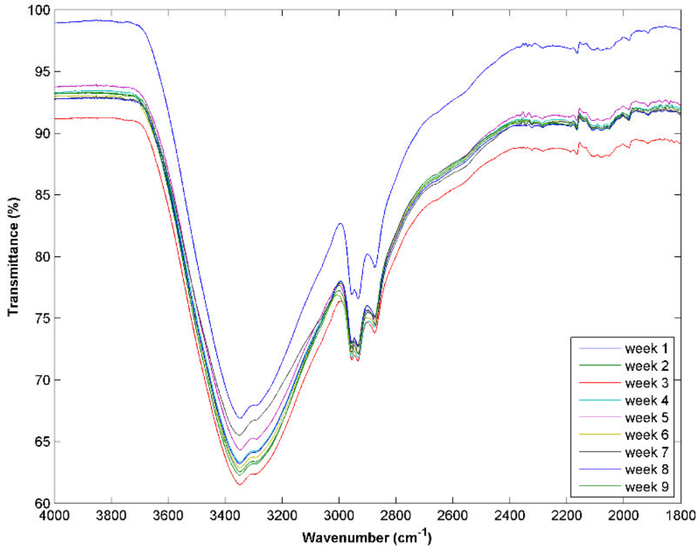

(a)

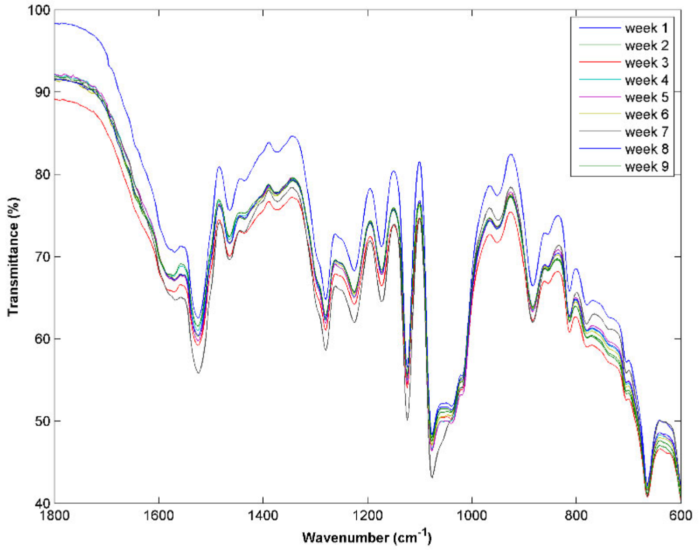

(b)

Figure *S2.* MIR spectra for the TLB-THEDES made for 9 weeks (once a week) to study its stability at room temperature **(a)** spectral range between 4000 cm^{-1} and 1800 cm^{-1}; **(b)** spectral range between 1800 cm^{-1} and 600 cm^{-1}.

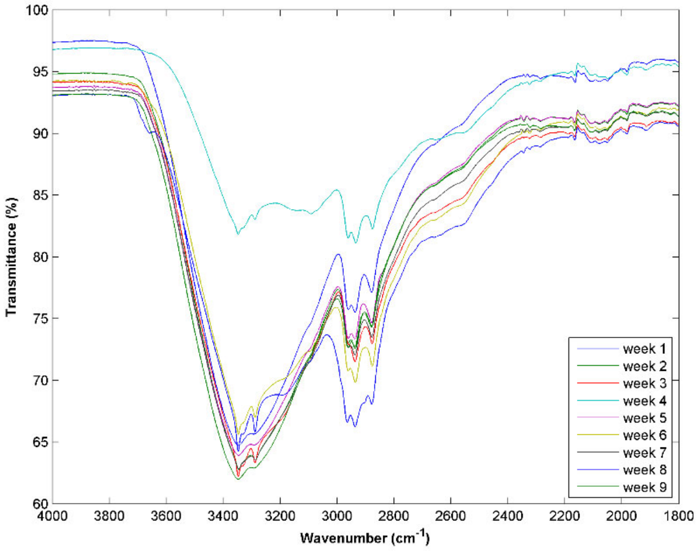

(a)

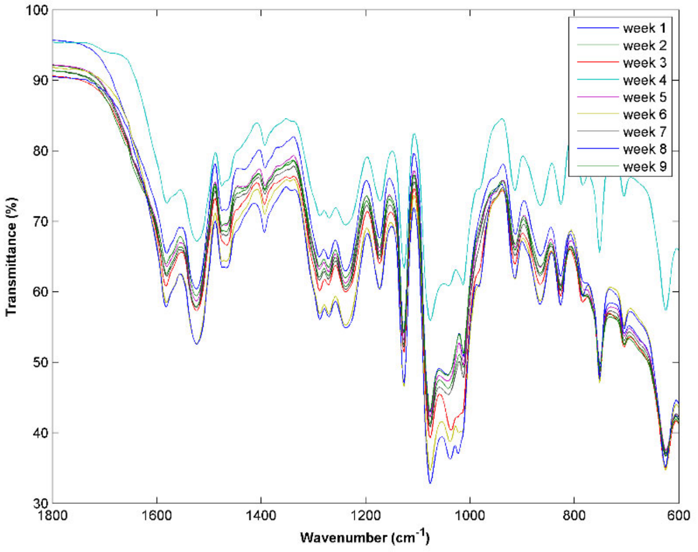

(b)

Figure *S3.* MIR spectra for the CLP-THEDESmade for 9 weeks (once a week) to study its stability at 40ºC **(a)** spectral range between 4000 cm^{-1} and 1800 cm^{-1}; **(b)** spectral range between 1800 cm^{-1} and 600 cm^{-1}.

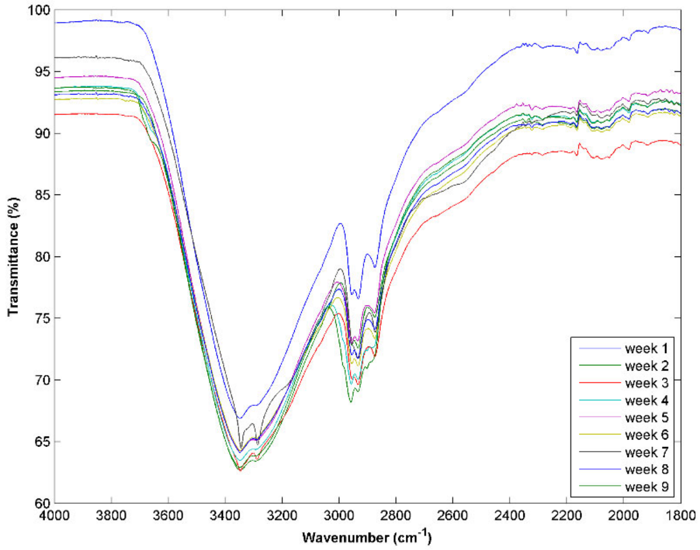

(a)

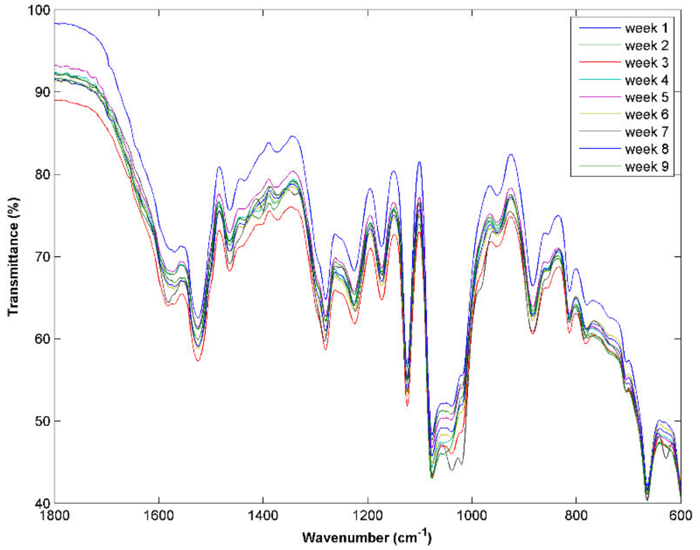

(b)

Figure *S4.* MIR spectra for the TLB-THEDES made for 9 weeks (once a week) to study its stability at 40ºC **(a)** spectral range between 4000 cm^{-1} and 1800 cm^{-1}; **(b)** spectral range between 1800 cm^{-1} and 600 cm^{-1}.
